# C-H Functionalization via Iron-Catalyzed Carbene-Transfer Reactions

**DOI:** 10.3390/molecules25040880

**Published:** 2020-02-17

**Authors:** Claire Empel, Sripati Jana, Rene M. Koenigs

**Affiliations:** Institute of Organic Chemistry, RWTH Aachen University, Landoltweg 1, D-52074 Aachen, Germany; claire.empel@rwth-aachen.de (C.E.); janasripati@gmail.com (S.J.)

**Keywords:** iron, carbene, diazoalkane, C-H functionalization

## Abstract

The direct C-H functionalization reaction is one of the most efficient strategies by which to introduce new functional groups into small organic molecules. Over time, iron complexes have emerged as versatile catalysts for carbine-transfer reactions with diazoalkanes under mild and sustainable reaction conditions. In this review, we discuss the advances that have been made using iron catalysts to perform C-H functionalization reactions with diazoalkanes. We give an overview of early examples employing stoichiometric iron carbene complexes and continue with recent advances in the C-H functionalization of C(sp^2^)-H and C(sp^3^)-H bonds, concluding with the latest developments in enzymatic C-H functionalization reactions using iron-heme-containing enzymes.

## 1. Introduction

**Scheme 1:**
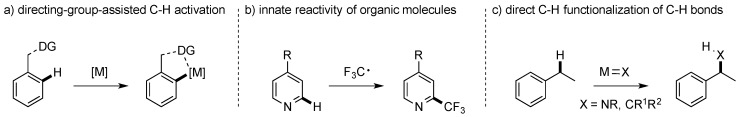
Strategies for the functionalization of C-H bonds. (**a**) directing-group-assisted C-H activation, (**b**) innate reactivity of organic molecules, (**c**) direct C-H functionalization of C-H bonds.

C-H bonds belong to the most common motifs in organic molecules and their direct functionalization is one of the main challenges in synthesis methodology, which impacts on the step economy and sustainability of chemical processes. In recent decades, this research area has flourished and different approaches have been realized to enable C-H functionalization reactions via different strategies: (a) by the directing group-assisted C-H activation, (b) via the innate reactivity of organic molecules, or (c) via direct C-H functionalization (Scheme 1) [1,2,3,4]. While the introduction and subsequent removal of directing groups is a prerequisite for directed C-H activation, direct C-H functionalization allows the introduction of new functional groups into organic molecules without the need for directing groups. It thus represents the most efficient and step-economic strategy by which to conduct C-H functionalization reaction. The challenge of this strategy lies within the selective activation of only one type of C-H bond; elegant methods have been developed in recent years, mainly relying on the use of expensive and mostly toxic precious metal catalysts based on rhodium, iridium, palladium, and others to direct the C-H functionalization reaction as either the catalyst or the substrate [5,6,7].

High costs of catalysts, limited resources, and toxicity of precious metals are the main drivers in the exploration of new synthesis methods based on third-row transition metals. Among these, iron plays a pivotal role. It is the second most abundant metal in the Earth’s crust following aluminum, and catalysts based on iron are currently emerging as new and important catalysts able to improve the environmental impact of chemical processes [8,9,10,11,12,13]. In nature, iron-containing enzymes play an important role, e.g., in the hemoglobin or myoglobin enzymes that are important for oxygen transport in mammals. They can also be found in cytochrome P450 enzymes or iron-sulfur clusters, amongst others, and are pivotal for the detoxification, e.g., by C-H oxidation reactions, of xenobiotics [14]. Against this background, the development of C-H functionalization reactions with iron catalysts has received significant attention over the years with the goal of reducing the economic and environmental footprint of organic synthesis methodologies, but also that of accessing new reactivity that is unique to iron. In this review, we discuss the advances made in this research area with a focus on C-H functionalization reactions with carbenes.

## 2. Iron in Carbene-Transfer Reactions

**Scheme 2:**
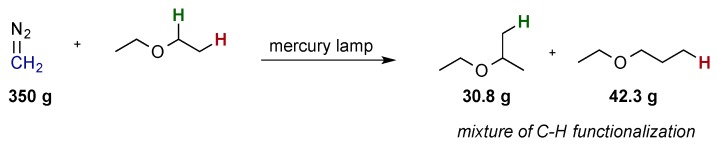
Photochemical C-H functionalization of diethyl ether with diazomethane.

Carbene-transfer reactions are one of the key strategies used today to conduct highly efficient and selective C-H functionalization reactions. The earliest examples date back to reports by Meerwein and Doering from 1942 and 1959, in which they described metal-free photochemical C-H functionalization reactions with diazoalkanes using high-energy UV light via free carbene intermediates (Scheme 2) [15,16]. However, the high reactivity of the free carbene intermediate led to unselective reactions and, as a consequence, a lack of applications in organic synthesis. In the subsequent decades, these shortcomings led to the development of metal-catalyzed carbine-transfer reactions using noble metals such as Rh(II), Ru(II), Ir(III), Au(I), Pd(II), or Cu(I) [17,18,19,20,21].

**Scheme 3:**
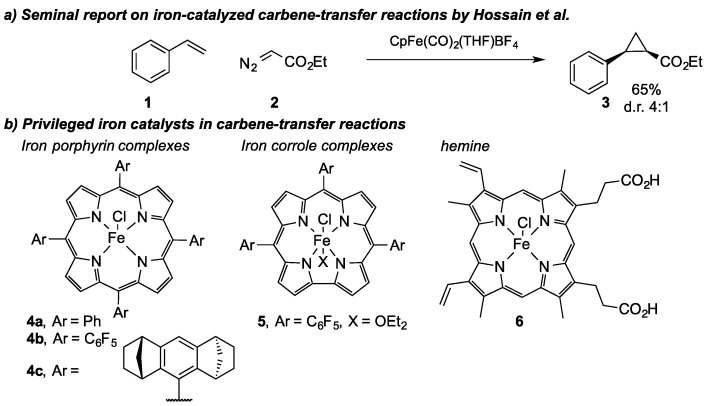
(**a**) Seminal iron-catalyzed cyclopropanation by Hossain et al. [22]. (**b**) Privileged iron catalysts in carbene-transfer reactions.

More recently, carbene-transfer reactions with iron complexes have gained significant attention in organic synthesis methodology for their potential to overcome the limitations of precious metal complexes. In 1992, Hossain et al. described the first iron-catalyzed carbene-transfer reaction using the cationic CpFe(CO)_2_(THF)BF_4_ complex in cyclopropanation reactions of styrene **1** and ethyl diazoacetate **2** (Scheme 3a) [22]. Following this strategy, different groups have since reported on their efforts to conduct iron-catalyzed carbene-transfer reactions [11,12,13].

In the following years, several groups reported their efforts in the field of iron-catalyzed carbine-transfer reactions. Woo et al. reported an important milestone in this research area when they uncovered the cyclopropanation of styrenes with ethyl diazoacetate **2** using an iron porphyrin complex (**4a**) in 1995 [23]. In this report and in further reports by the Che (**4c**) and Aviv (**5**) groups, the fine-tuning of iron porphyrin complexes by the axial ligand and/or electronics of the porphyrin ring system was demonstrated (Scheme 3b) [24,25,26]. Today, iron porphyrin complexes and derivatives thereof are important cheap and readily available catalysts to enable carbine-transfer reactions under mild conditions.

**Scheme 4:**
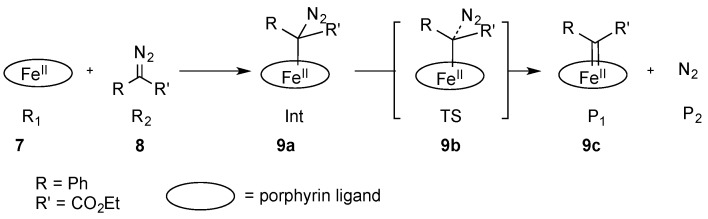
Mechanism of the formation of iron carbene complexes.

Khade and Zhang studied the formation of iron carbene complexes via quantum chemical studies using ethyl phenyldiazoacetate (**8**) as a model compound. They were able to identify a reaction intermediate (**9a**) in which ethyl phenyldiazoacetate (**8**) coordinated to the iron porphyrin complex **7** via the carbon atom. In a next step, nitrogen was expelled via transition state **9b** and the iron carbene complex **9c** was formed. In these studies, the authors were also able to show that *N*-methyl imidazole reduced both the reaction energy ΔG and the activation energy ΔG_TS_ (Scheme 4) [27].

## 3. Stoichiometric C-H Functionalization

**Scheme 5:**
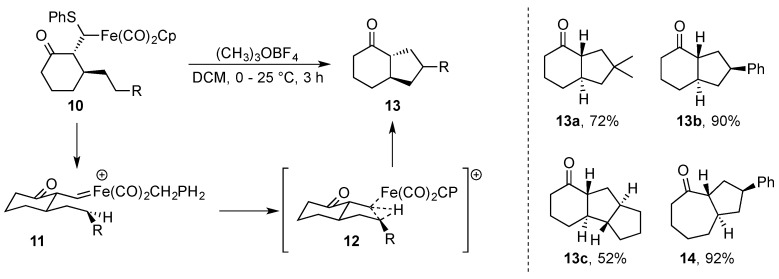
Intramolecular C-H insertion reactions for the synthesis of cyclopentane derivatives (**13**,**14**) by Helquist and coworkers [29,30].

Initial applications of iron carbene complexes in C-H functionalization reactions involved the use of preformed iron carbene complexes **10** that were used in intramolecular C-H insertion reactions. The nature of the preformation of the iron carbene complex renders these applications stoichiometric in iron. Following this methodology, C-H insertion occurs selectively to form cyclopentane ring systems (**13**,**14**). The corresponding bi- and tricyclic target structures can be obtained with high *trans*-selectivity (Scheme 5) [28]. The same authors investigated the selectivity of this process in further studies and explained the observed reaction outcome via a concerted C-H insertion reaction that proceeds via a chair-like cyclic transition state (**12**, Scheme 5) [29,30].

## 4. C-H Functionalization Reactions of Aromatic C-H Bonds

**Scheme 6:**
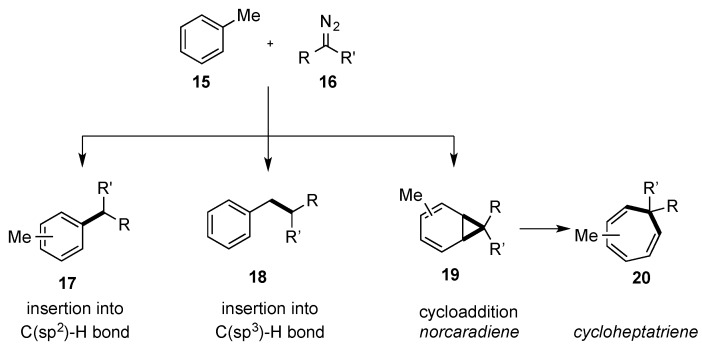
Possible products in the iron-catalyzed functionalization of toluene **15** by carbene insertion.

The direct C-H functionalization of benzene or alkyl benzenes (**15**) via carbene or metal-carbene intermediates can give access to three different products, which arise from (a) insertion into a C(sp^2^)-H bond (**17**), (b) insertion into a side-chain C(sp^3^)-H bond (**18**), or (c) a cycloaddition reaction with the aromatic system to give a norcaradiene **19** that can undergo a Buchner reaction to give a cycloheptatriene **20** (Scheme 6). The challenge lies in the chemoselective differentiation of these three reaction pathways. Moreover, a C-H insertion into a C(sp^2^)-H bond may give rise to different regioisomers.

**Scheme 7:**
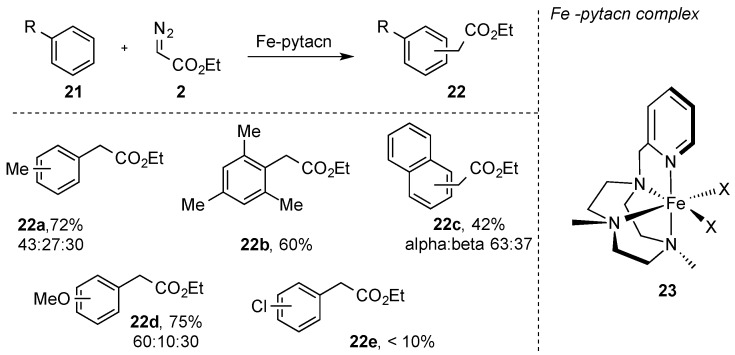
(**Left**): functionalization of arenes **21** with ethyl diazoacetate **2**; (**Right**): iron-pytacn complex.

In this context, Luis, Perez, and coworkers reported on the use of iron(II)-complex (**23**) bearing a pytacn ligand in the reaction of ethyl diazoacetate **2** with aromatic hydrocarbons (**21**). Under these reaction conditions, ethyl diazoacetate (**2**) underwent a chemoselective C-H functionalization of the aromatic C(sp^2^)-H bond of benzene and alkyl benzene derivatives, and only trace amounts of the norcaradiene/cycloheptatriene reaction pathway were observed [31]. The substrate scope of benzene derivatives was further studied by the same authors and, in all cases, exclusive C(sp^2^)-H bond insertion occurred in moderate to good yields as a mixture of regioisomers. Electron-withdrawing groups (e.g., Cl) had a detrimental effect on the C-H functionalization reaction, and the corresponding products were obtained with reduced yields (Scheme 7) [32].

**Scheme 8:**
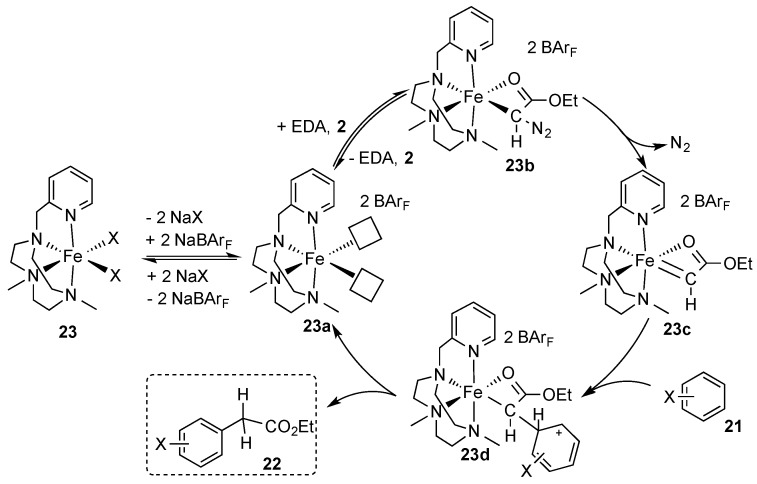
Proposed reaction pathway for the C-H functionalization reaction of arenes (**21**) using ethyl diazoacetate **2**.

The reaction mechanism of this reaction was studied in detail by the authors. In a first step, the iron complex **23** undergoes a counterion exchange with NaBAr_F_ to give the dicationic complex **23a**. The latter undergoes formation of the initial coordination of ethyl diazoacetate (**23b**), which leads to the formation of an iron carbene complex **23c** upon extrusion of nitrogen. Next, the arene **21** undergoes nucleophilic addition to **23c** under formation of a Wheland-type intermediate **23d**, which undergoes a 1,2-H shift to release product **22** and the active catalyst **23a** (Scheme 8) [32]. The proposed mechanism was in line with a previous report by Doyle, Padwa, and coworkers [33].

**Scheme 9:**
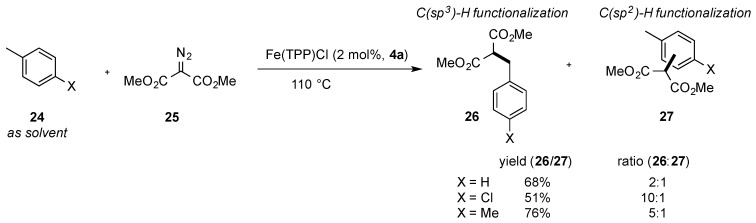
Chemoselectivity between C(sp^2^)-H bond and C(sp^3^)-H bond insertion reaction investigated by Woo and coworkers [34].

The direct C-H functionalization reaction of aromatic compounds (used as solvents) was also studied by Woo and coworkers using dimethyl diazomalonate **25** and an iron(III) porphyrin complex (**4a**) as a catalyst at elevated reaction temperatures. However, only moderate chemoselectivity of C(sp^2^)-H bond vs. C(sp^3^)-H bond insertion could be achieved in this reaction (**26**:**27**, 2:1 to 10:1). Different substituted aromatic systems such as toluene, mesitylene, 4-chloro-toluene, or anisole were shown to be compatible with the present reaction conditions. In contrast to the previously described reaction, the product from the C(sp^3^)-H bond insertion reaction was obtained as the major product (Scheme 9) [34].

**Scheme 10:**
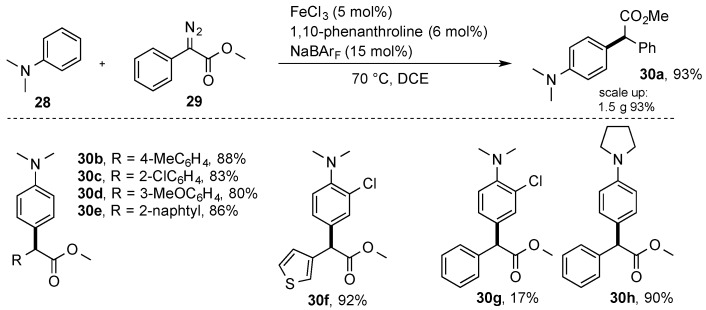
Iron-catalyzed arylation of donor-acceptor diazo compound **29** with *N*,*N*-dialkyl anilines **28** by the Zhou group [36].

In 2015, Zhou and coworkers studied the reaction of donor–acceptor diazo compounds **29** with electron-rich aromatics **28**. Using a simple, non-porphyrin iron complex, the authors were able to demonstrate the C(sp^2^)-H functionalization reaction of *N*,*N*-dialkyl aniline derivatives (**28**) with high yield and selectivity. In all cases, selective C-H functionalization in the 4 position occurred, which could be rationalized by the high nucleophilicity of *N*,*N*-dialkyl aniline derivatives in the *para* position. From the viewpoint of the catalyst, a very simple iron catalyst was used that could be obtained in situ from FeCl_3_, 1,10-phenanthroline, and NaBAr_F_ (Scheme 10) [35]. More recently, Deng and coworkers reported on a similar transformation using a *bis*(imino)pyridine iron complex, which could be used in the direct C-H functionalization of *N*,*N*-dimethylaniline **28** to give **30a** with a 71% yield [36].

## 5. C-H Functionalization Reactions of Heteroaromatic C-H Bonds

*N*-heterocycles like indoles or pyrroles are privileged motifs in drugs and natural products, and a broad variety of strategies for their construction or their functionalization has been described in recent decades. A particularly intriguing transformation is the direct C-H bond functionalization of heterocycles, which would streamline current synthesis strategies of drugs or natural products. Furthermore, it would also allow the introduction of molecular diversity into late-stage functionalization reactions. This approach, in combination with the benefits of iron catalysts, would enable important applications of C-H functionalization strategies in medicinal chemistry, agrochemistry, or total synthesis.

**Scheme 11:**
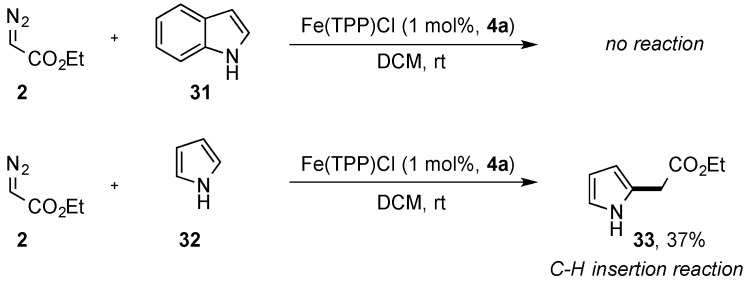
Iron-catalyzed reaction of ethyl diazoacetate **2** with indole **31** and pyrrole **32**.

In 2006, Woo et al. reported on the iron-catalyzed functionalization of *N*-heterocycles with ethyl diazoacetate **2** as a carbene precursor. While no reaction was observed with indole **31**, the closely related pyrrole **32** underwent C-H functionalization without concomitant N-H functionalization to give **33** in a 37% yield (Scheme 11) [37].

**Scheme 12:**
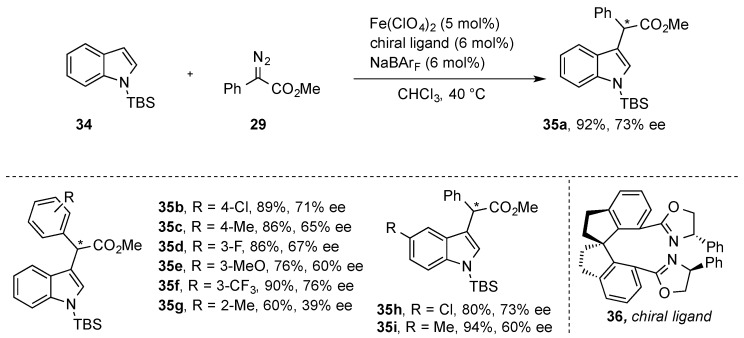
Enantioselective iron-catalyzed C-H functionalization of TBS-indole (**34**) with α-aryl-α-diazoesters (**29**), described by Zhou et al. [38].

Thereafter, Zhou and-coworkers reported on an enantioselective C-H functionalization of indole heterocycles. For this purpose, they studied the reaction of silyl-protected indole heterocycles (**34**) with α-aryl-α-diazoesters (**29**) in the presence of Fe(ClO_4_)_2_ and a chiral spirobisoxazoline ligand **36**. This strategy allowed the C-H functionalization of indole heterocycles under mild reaction conditions with moderate enantioselectivity. Importantly, different substituents at the indole heterocycles were tolerated, such as chlorine and methyl (Scheme 12) [38].

**Scheme 13:**
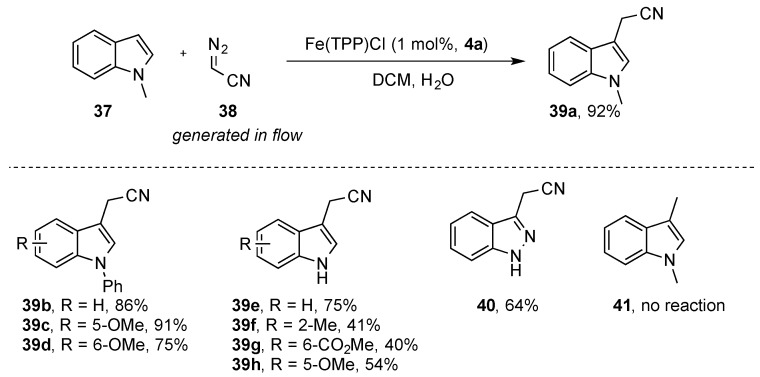
Reaction of diazoacetonitrile **38** with indole heterocycles using Fe(TPP)Cl, reported by Koenigs, Weissenborn, and coworkers [39].

As described by Woo and coworkers, the direct functionalization of indole heterocycles in the C3 position does not readily occur when using ethyl diazoacetate [37]. The direct functionalization of indole with acceptor-only diazoalkanes would open up avenues towards the efficient synthesis of tryptamines and related alkaloids. In recent years, different groups have focused their efforts on identifying iron catalysts and/or reaction conditions that would allow this important transformation. In 2019, Koenigs and Weissenborn et al. reported the iron-catalyzed C-H functionalization reaction of indoles **37** in the C3 position using the same catalyst as the Woo group, but using diazoacetonitrile **38**. Using this methodology, protected and unprotected indole and indazole heterocycles underwent selective functionalization in the C3 position. Importantly, no reaction was observed when blocking the C3 position (Scheme 13). This approach has now enabled a two-step approach towards the synthesis of important tryptamine derivatives [39].

**Scheme 14:**
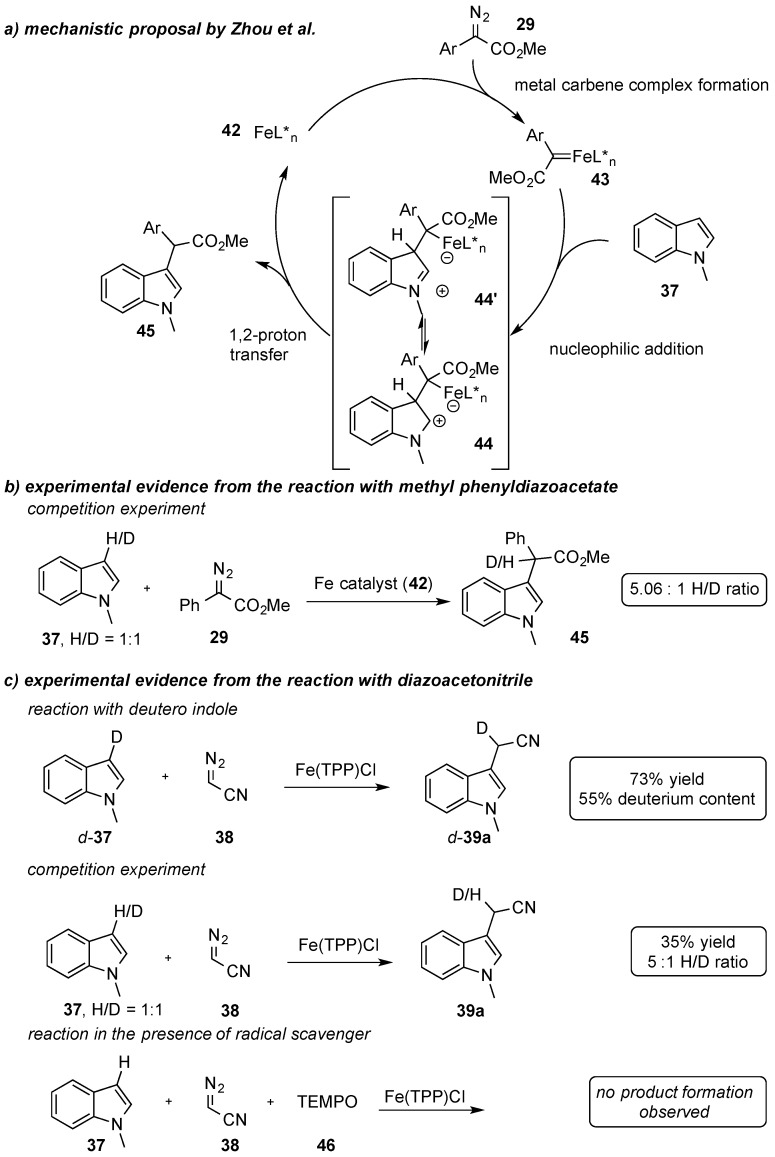
Hypothesized mechanism for the C-H functionalization of indole with iron carbene complexes and experimental evidence. (**a**) mechanistic proposal by Zhou et al., [38], (**b**) experimental evidence from the reaction with methyl phenyldiazoacetate [38], (**c**) evidence from the reaction with diazoacetonitrile [39].

To date, various proposals for the C-H functionalization of indole heterocycles have been reported in the literature. One important mechanistic proposal suggests the initial formation of an iron carbene complex **43** that undergoes nucleophilic addition of an indole followed by a [1,2]-proton transfer reaction (Scheme 14a). Zhou and coworkers reported a KIE (= kinetic isotope effect) of 5.06 in the reaction of *N*-methyl indole and deuterated *N*-methyl indole, which led them to conclude a proton-transfer reaction to be the rate-determining step (Scheme 14b) [38]. Koenigs, Weissenborn, and coworkers observed a similar trend in the reaction of *N*-methyl indole and deuterated *N*-methyl indole with diazoacetonitrile using both a synthetic and an enzymatic iron catalyst. However, investigations using TEMPO **46** as a radical scavenger revealed a complete inhibition of the C-H functionalization reaction. Based on these experimental data, the authors assumed a radical reaction, yet it was not yet clear in which particular reaction step radical intermediates were involved (Scheme 14c) [39].

The reaction mechanism of the C-H functionalization reaction of indole heterocycles with iron carbene intermediates still remains unclear. It would be valuable to investigate this reaction via DFT calculations to gain knowledge of the exact spin state of the participating iron catalyst and to identify the exact reaction mechanism.

## 6. C-H Functionalization Reactions of Aliphatic C-H Bonds

The direct C-H functionalization reaction of aliphatic C-H bonds is an important strategy for the introduction of new functional groups onto a hydrocarbon skeleton; the difficulties lie in the differentiation of chemically very similar C-H bonds. Over the years, metal-catalyzed insertion reactions of carbene fragments have emerged as a promising strategy for this purpose, and a variety of precious-metal-catalyzed C(sp^3^)-H bond functionalization reactions have been described in the literature, ranging from site-selective C-H functionalization to late-stage functionalization of complex molecules [5,40,41].

**Scheme 15:**
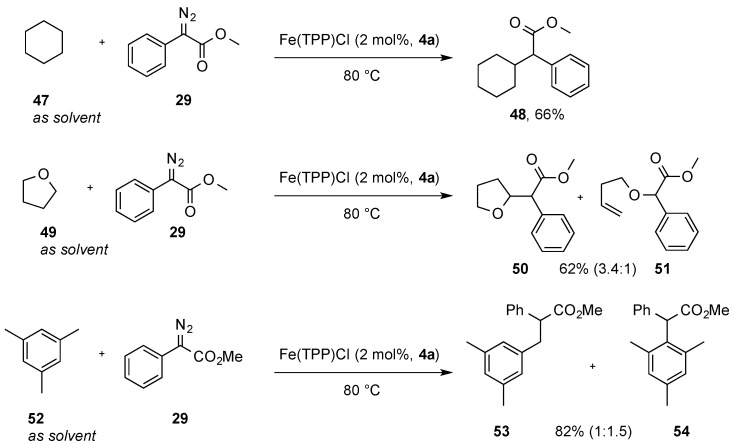
Reaction of α-aryl-α-diazoesters with cyclohexane (**47**), THF (**49**), and mesitylene (**52**).

The application of non-toxic and highly active iron catalysts has blossomed in the last few years, and today, iron-catalyzed carbine-transfer reactions have emerged as an important, environmentally benign strategy by which to conduct C(sp^3^)-H bond functionalization reactions. In this context, Woo and coworkers reported their studies in C-H functionalization reactions of cyclohexane **47** and tetrahydrofuran **49** as substrates. Using a donor–acceptor diazoalkanes (**29**) as a carbene precursor and a Fe(TPP)Cl (**4a**) catalyst, the authors demonstrated proof-of-concept studies in this research area. Cyclohexane **47** underwent smooth C-H functionalization to give product **48** with a 66% yield. No C-H functionalization was observed when ethyl diazoacetate **2** or dimethyl diazomalonate **25** were used as carbene precursors. Similarly, tetrahydrofuran **49** underwent selective C-H functionalization reaction in the β position (**50**). A byproduct arising from ring-opening of the THF ring was observed as a minor reaction product **51** with ~20% yield. In a competition experiment with mesitylene (**52**) as a substrate, the authors obtained the product from C(sp^3^)-H bond and C(sp^2^)-H bond in a 1.5:1 ratio (Scheme 15) [34].

**Scheme 16:**
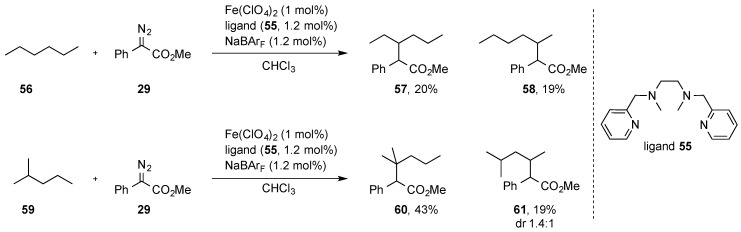
Studies on the selectivity of iron-catalyzed C(sp^3^)-H insertion reactions.

The C-H functionalization of cyclohexane represents one of the simplest examples, as all C-H bonds within cyclohexane are identical. Contrarily, linear or branched hydrocarbons are more challenging, as different C-H bonds are present that need to be differentiated by the catalyst. Zhu et al. studied the reaction of these substrates with donor–acceptor diazoalkanes using an iron catalyst. When *n*-hexane **56** was used as a substrate, selective C-H functionalization of secondary C-H groups occurred, but without any meaningful selectivity of the C2 vs. C3 positon. When studying 2-metylpentane **59**, the C-H functionalization occurred preferentially at the tertiary C-H group. No C-H functionalization of primary C-H bonds was observed, and the reactivity order for the C-H functionalization decreased from tertiary > secondary > allylic/benzylic >> primary C-H bonds (Scheme 16). It is of note that this catalyst can be applied in the C-H functionalization of cyclohexane with turnover numbers of up to 690 on gram scale. In this reaction, the authors observed a KIE of 2.0 for k_H_/k_D_ [42].

**Scheme 17:**
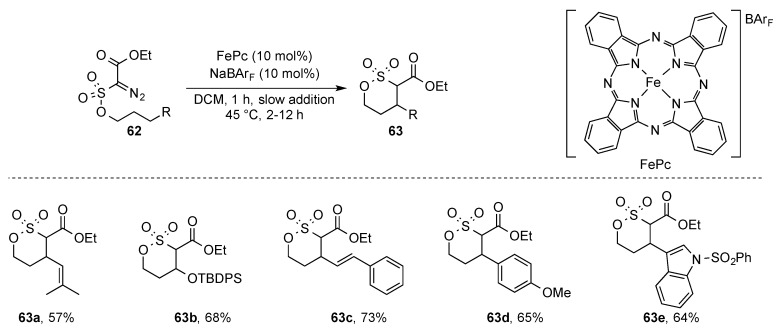
Intramolecular cyclisation via C-H functionalization for the synthesis of sulfonate esters.

While intermolecular C(sp^3^)-H functionalization reactions suffer from missing selectivity and reactivity, the development of intramolecular processes would enable important steps to be taken in the understanding of iron-catalyzed C(sp^3^)-H functionalization. In this context, the White group explored an intramolecular cyclization via C-H functionalization using an iron phthalocyanin complex as the carbene-transfer catalyst; importantly, weakly coordinating counterions needed to be used to increase the electrophilicity of the iron complex. Under these conditions, the *bis*-acceptor diazoalkanes (**62**) underwent smooth intramolecular C-H functionalization reaction to yield product **63** in moderate to good yields with broad functional group tolerance (Scheme 17). It is important to note that the C-H functionalization reaction selectivity occurred in the allylic, benzylic position or in the α position to a heteroatom [43].

**Scheme 18:**
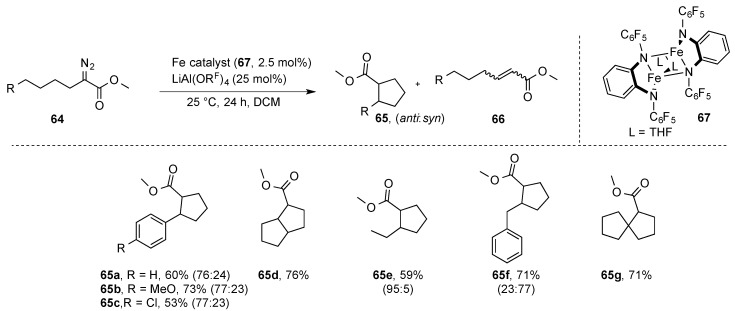
Intramolecular alkylation reaction for the synthesis of five membered rings.

Recently, Costas and coworkers reported on the intramolecular functionalization of C(sp^3^)-H bonds using the electrophilic [Fe(^F^pda)-(THF)]_2_ complex (**67**) as catalyst and a lithium salt with a weakly coordinating counterion as a co-catalyst. Importantly, the lithium salt is required to activate the diazoester under mild conditions, while the iron complex is needed for the actual carbene-transfer reaction. Following this strategy, intramolecular C-H functionalization reactions of C(sp^3^)-H could be realized to selectively obtain the five membered ring products (**65**). Following this strategy, bi- and spirocyclic systems were synthesized (Scheme 18). Under these conditions, the five membered ring was is favored, e.g., over benzylic C-H functionalization (**65f**). The formation of α,β-unsaturated esters (**66**) occurred via a β-hydride elimination reaction [44].

## 7. Biocatalytic C-H Functionalization Reactions of Aromatic C-H Bonds

Over the years, the development of non-natural activity of enzymes has developed as an important strategy by which to conduct highly efficient carbene-transfer reactions [45,46]. In this section, we have focused on the recent developments in this research area, with a focus on applications in C-H functionalization reactions.

**Scheme 19:**
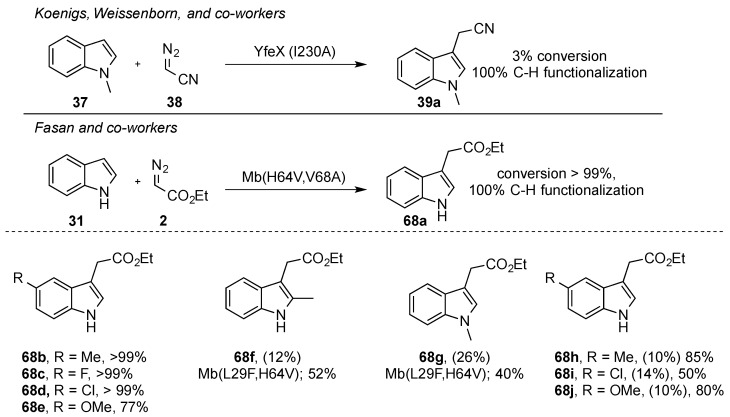
Enzyme-catalyzed C3-alkylation reaction of indoles.

In the report of Koenigs, Weissenborn, and coworkers, the reaction of *N*-methyl indole (**37**) with diazoacetonitrile (**38**) was also studied with the bacterial dye-decolorizing peroxidase YfeX from *E. coli*. A library of YfeX variants and the wild-type protein were studied and, under the best conditions, a turnover number of 37 was achieved with the wild-type enzyme. This TON (= turnover number) could be improved to 80 by using the I230A variant of YfeX. This variant was also studied in the reaction of *N*-methyl indole with ethyl diazoacetate **2**, which led to an increased TON of 236 (Scheme 17) [39]. At the same time, the Fasan group reported on the application of an engineered myoglobin enzyme in the C3-functionalization reaction of unprotected indoles using ethyl diazoacetate **2** as a carbene precursor. The Mb(H64V,V68A) variant gave the highest conversion and a TON of 82 in a whole-cell setup, and **68a** was obtained as the only product of C-H functionalization. In their report, Fasan and coworkers also investigated the functional group tolerance of this reaction, and different substitution patterns on the indole heterocycle were tolerated. Notably, in the case of *N*-protected indoles, the Mb(L29F,H64V) variant was required to achieve a similar activity of the enzyme (Scheme 19) [47].

**Scheme 20:**
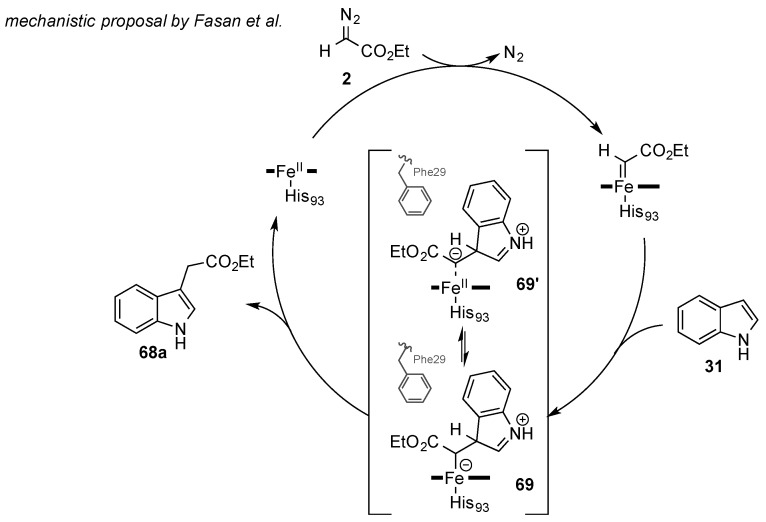
Mechanistic investigations and proposed catalytic cycle by the Fasan group.

Fasan and coworkers studied the reaction mechanism of the enzymatic C-H functionalization reaction. For this purpose, the reaction of 3-deutero-indole ***d*-31** and ethyl diazoacetate **2** was investigated. The C-H functionalization product **68a** was obtained as the protonated product exclusively, and no deuterium label was found in the reaction product. Moreover, in a competition experiment, no difference in reaction kinetics of the deuterated and non-deuterated starting material was observed. The absence of a kinetic isotope effect led the authors to the conclusion that no C-H insertion mechanism occurred. Based on this evidence, Fasan and coworkers concluded a nucleophilic attack by the indole substrate **31** to generate a zwitterionic intermediate **69**/**69′**, which underwent a solvent-assisted proton-transfer reaction to give the desired C-H functionalization product (Scheme 20) [47]. At this point, it is noteworthy that Koenigs and Weissenborn et al. were able to observe a retention of the deuterium label in the reaction of deuterated *N*-methyl indole *d*-**47** with diazoacetonitrile [39].

**Scheme 21:**
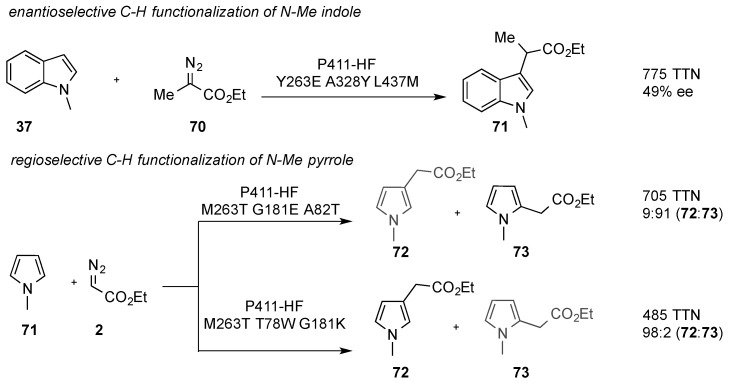
Indole C3-alkylation with engineered P411-HF enzymes; regioselective alkylation of 1-methylpyrrole **53**.

Shortly after the reports by Koenigs, Weissenborn. and Fasan, the enzymatic C-H functionalization of indole and other heterocycles with an engineered cytochrome P411 enzyme was studied by Arnold and coworkers. They reported on the directed evolution of a “carbene transferase” enzyme that enables the highly efficient, chemoselective, and biocatalytic C-H functionalization of indole and pyrrole heterocycles. Using a UV-Vis spectrophotometry-based high-throughput screening, the authors were able study several thousand mutants to engineer enzymes to conduct chemoselective C-H functionalization reactions. Cytochrome P411 variants were tested in reactions with *N*-Me pyrrole (**71**), and two different variants allowed the regioselective C-H functionalization in the C3 position or the C2 position as well as enantioselective C-H functionalization of indole heterocycles with diazopropionate (Scheme 21) [48].

**Scheme 22:**
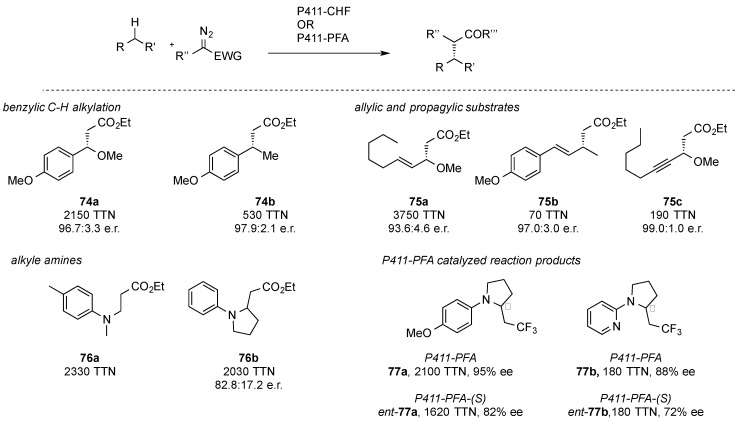
C-H functionalization using engineered P411-CHF and P411-PFA enzymes.

In further reports, the Arnold group studied engineered cytochrome P411 enzymes in benzylic C-H functionalization reactions of alkyl arenes with ethyl diazoacetate (**2**). A cytochrome P411 variant with an axial serine ligand served as the starting point for the directed evolution, which led to the P411-CHF variant that gave a TTN (= total turnover number) of 2020 and an enantiomeric ratio of 96.7:3.3 (**74a**, Scheme 22). In further studies, the C-H functionalization of allylic and propargylic substrates as well as alkyl amines was studied (**75**, **76**, Scheme 22) [49]. Only recently, Arnold et al. were able to further extend the substrate scope of carbene precursors to trifluoro diazoethane, which was studied in the α-C(sp^3^)-H functionalization of N,N-dialkyl aniline derivatives. By directed evolution of the wild-type cytochrome P411 enzyme the C-H functionalization reaction with trifluoro diazoethane was realized. Further studies focused on the access of the opposite stereoisomer. Starting from a P411 variant that provided the opposite stereochemistry, direct evolution led to a variant providing the inverse stereochemistry. This example showcases the power of biocatalytic transformations and the fact that both enantiomers of a desired product can be obtained by means of directed evolution (**77**, Scheme 22) [50].

## 8. Conclusions and Perspectives

Over the years, the application of iron complexes has gained significant attention for the conduction of C-H functionalization reactions with diazoalkanes under mild and sustainable reaction conditions. A variety of iron complexes have been described to date to perform C-H functionalization reactions with high efficiency, which have leveraged iron-catalyzed carbine-transfer reactions as an important tool for organic chemists to functionalize unreactive C-H bonds. In light of the recent developments in enzyme-catalyzed carbene-transfer reactions and directed evolution, iron-heme enzymes are now developing as important tools with which to conduct highly chemo-, regio-, and stereoselective C-H functionalization reactions. Building upon these advances, future developments of iron-catalyzed C-H functionalization might include, for example, the site-selective activation of unactivated C(sp^3^)-H bonds, the broadening of the substrate scope to include non-activated aromatic systems, or development of an understanding of the reaction mechanisms and the spin state of iron within the catalytic cycle.
